# Ambient air pollutants, increased anaemia risk, and vulnerable subgroups: evidence from a large group of workers in South China

**DOI:** 10.7189/jogh.15.04346

**Published:** 2025-12-19

**Authors:** Xinyue Li, Zhishen Wu, Yanjie Zhao, Xudan Chen, Zhiqiang Li, Yongqing Sun, Yajun Gong, Peixia Hu, Xiangyuan Huang, Weiyi Pan, Shen Xie, Wangjian Zhang, Yongshun Huang

**Affiliations:** 1 Department of Medical Statistics, School of Public Health, Sun Yat-sen University, Guangzhou, China; 2 Guangdong Province Hospital for Occupational Disease Prevention and Treatment, Guangzhou, China; 3 Stomatological Hospital, Southern Medical University, Guangzhou, China; 4 Center for Clinical Big Data and Analytics Second Affiliated Hospital, Department of Big Data in Health Science School of Public Health, Zhejiang Key Laboratory of Intelligent Preventive Medicine, Zhejiang University School of Medicine, Hangzhou, Zhejiang, China; 5 Qingdao Hospital, University of Health and Rehabilitation Sciences (Qingdao Municipal Hospital), Qingdao, Shandong, China; 6 Department of Ultrasound, Beijing Obstetrics and Gynecology Hospital, Capital Medical University, Beijing Maternal and Child Health Care Hospital, Beijing, China; 7 School of Public Health, Southern Medical University, Guangzhou, China; 8 School of Public Health, Shanxi Medical University, Taiyuan, China; 9 Department of Public Health and Preventive Medicine, School of Medicine, Jinan University, Guangzhou, China

## Abstract

**Background:**

Previous epidemiological studies indicated a potential correlation between air pollution and anaemia, particularly in children, pregnant women, and the elderly. However, evidence is insufficient for workers exposed to air pollution while working in environments with other occupational hazards. Based on data from a substantial population of workers in southern China, we aimed to examine the relationships between different air pollutants and both haemoglobin (Hb) concentration and the prevalence of anaemia.

**Methods:**

In this cross-sectional analysis, we recruited 372 290 workers from the National Occupational Disease Surveillance Platform and utilised a mixed-effects model to explore the association of various air pollutants (including PM_2.5_, PM_10_, PM_coarse_, O_3_, and NO_2_) with Hb concentration and the prevalence of anaemia. We ran stratified analyses by various demographic characteristics and occupational variabels.

**Results:**

Each 5 μg/m^3^ increase in the concentration of PM_2.5_, PM_10_, PM_coarse_, O_3_, and NO_2_ corresponds to a 2.037 g/L (95% confidence interval (CI) = 1.938, 2.137), 1.096 g/L (95% CI = 1.040, 1.152), 1.412 g/L (95% CI = 1.313, 1.510), 0.518 g/L (95% CI = 0.489, 0.547), and 0.250 g/L (95% CI = 0.219, 0.281) decrease in Hb concentration, respectively. The prevalence of anaemia increased by 11.3% (95% CI = 7.3, 15.5), 5.0% (95% CI = 2.8, 7.3), and 4.5% (95% CI = 6, 8.5) for a 5 μg/m^3^ increase in PM_2.5_, PM_10_, and PM_coarse_, respectively, with the impact being more pronounced in the non-benzene-exposed population. Subgroup analyses suggested potential variations in susceptibility to the same air pollutant across different demographics and occupational variables.

**Conclusions:**

The Hb levels among the workers in our sample were associated with various atmospheric pollutants, with certain demographic and occupational subgroups being particularly vulnerable. These results highlight the need for targeted air pollution control and occupational health interventions, particularly for vulnerable demographic and occupational subgroups.

There were approximately 1.8 billion cases of anaemia globally in 2019, contributing to about 50.3 million disability-adjusted life years [1]. In China, which represented over 18% of the global population at the time, the prevalence of anaemia among residents aged 18 years and above was 8.7% [2]. The condition manifests as a decline in red blood cell count, size, or haemoglobin (Hb) concentration, which impairs the oxygen-carrying capacity of the affected individual’s blood [3]. Due to physiological differences, anaemia is more common among susceptible groups such as children, women of reproductive age, and older adults [4]. However, the prevalence of the condition among workers has been overlooked in existing research. One systematic review examining the impact of anaemia on occupational labor productivity found that it contributed to increased fatigue and that, if left untreated, it could lead to severe complications, thereby incapacitating individuals from engaging in work [5].

Air pollution also poses a significant threat to both global health and economic prosperity: it was responsible for 74.4% of pollution-related deaths and accounted for losses equivalent to 6.1% of global economic output in 2019 [6]. Importantly, previous studies have indicated a possible association between air pollution and anaemia [7]. In two studies focussing on elderly populations in China and the USA, exposure to PM_2.5_ and NO_2_ was correlated with decreased Hb concentration and a higher prevalence of anaemia [8,9]. Similarly, according to a retrospective cohort study conducted in China among 7932 pregnant women, ambient PM_2.5_ exposure and its chemical components were linked to lower Hb concentrations [10]. Research from Peru showed that exposure to PM_2.5_ was linked to moderate/severe anaemia among children aged 6–59 months [11]. However, there is currently a lack of evidence on the existence of these associations among workers – a key group for economic activities in countries worldwide. This population is often exposed to hazardous materials (*e.g.* lead, benzene, arsenic, radioactivity) that, along with air pollution, can have additive, synergistic, or antagonistic effects for their haematological health. For instance, air pollution may disrupt the hepcidin-iron transporter axis via immune activation and inflammation, leading to iron imbalance, lower Hb concentration, and increased anaemia risk [12,13]. Occupational factors such as lead, benzene, arsenic, and radioactive substances could also cause the development of anaemia in exposed individuals. The underlying mechanisms include the inhibition of key enzymes in haeme synthesis, disruption of transcriptional regulation in erythropoiesis, sustained hematopoietic suppression, and perturbations in iron metabolism within the bone marrow [14–17]. Despite this, existing studies mainly focussed separately on the health effects of air pollution and occupational hazards, rather than their interactive effects.

We set out to explore how different air pollutants relate to Hb concentration and anaemia prevalence in a substantial population of workers from southern China, and to identify potential modifying effects of demographics and occupational factors on these associations.

## METHODS

### Study design and population

We obtained the data for this cross-sectional study from the National Occupational Disease Surveillance Platform, which contains the health record data of workers in Guangdong Province from 1 January 2020 to 31 December 2020, including demographic variables such as age, gender, occupational variables, and laboratory measures like Hb concentration. A stratified multi-stage random sampling method was employed to select individuals from 21 cities in the Guangdong area. Outcome data within the Platform (*e.g.* Hb levels) were collected by medical personnel from government-certified occupational health examination institutions. Occupational variables, including length of service, were registered and reported by the respective employing companies. All data were collected in 2020, underwent a standardised verification process prior to database entry, and no repeated measurements were present. Consequently, each observation (row) in the final data set represents a unique and independent worker, ensuring the independence of all data points.

### Outcome measurement and definition

Our primary outcomes of interest were Hb concentration and anaemia prevalence. Based on the World Health Organization (WHO) guidelines and the diagnostic standards for anaemia in the Chinese population, we defined anaemia as a Hb level <120g/L for adult males and <110g/L for adult females [18]. According to WHO guidelines [18] and the Chinese health industry standard (WS/T 441-2013) [19], altitude adjustment of Hb thresholds is generally unnecessary for diagnosing anaemia in regions below 1000 m. As most participants in our study resided in areas with altitudes below 500 m [20], we applied fixed Hb thresholds without altitude-based correction. To measure the concentration of Hb within the Platform, fasting blood samples were collected using anticoagulant tubes, which were then mixed by inverting them 3–5 times to ensure proper mixing. The Hb level was analysed using an automated blood cell analyser, which employed a colorimetric method to accurately measure the concentration.

### Exposure data simulation

We retrieved relevant air pollution indicators from the China High Air Pollutants data set, the detailed simulation procedures of which can be found elsewhere [21–25]. Briefly, the CHAP data set used a space-time extremely randomised trees model that captured spatiotemporal variations, elucidating heterogeneity and confirming the reliability and applicability of the pollutant estimates. The reported cross-validation coefficients of determination for daily predictions were 0.86–0.90 for PM_2.5_, 0.83–0.87 for PM_10_, 0.87 for O_3_, and 0.93 for NO_2_, with corresponding root-mean-square error values of 10.0–18.4 μg/m^3^, 19.7–28.4 μg/m^3^, 17.10 μg/m^3^, and 4.89 μg/m^3^, respectively. Here, we obtained PM_2.5_, PM_10_, PM_coarse_ (coarse particulate matter, with aerodynamic diameter between 2.5 and 10μm, calculated as the arithmetic difference between the model-estimated PM_10_ and PM_2.5_ concentrations at the same location), and NO_2_ data at a spatial resolution of 1 km ×1 km, while O_3_ data had a 10 km ×10 km resolution. All pollutants had daily temporal resolution. We used nearest-distance matching method to determine pollutant concentrations near each participant's workplace and calculated the cumulative average exposures for the year preceding their physical examination.

Moreover, we sourced data on annual temperature (°C) and relative humidity (%) from the National Earth System Science Data Center, National Science & Technology Infrastructure of China [26]. These data, recorded monthly at the 1 km^2^ resolution, were averaged annually and assigned to participants.

### Statistical analysis

We constructed the generalised linear mixed-effects models to examine the impact of PM_2.5_, PM_10_, PM_coarse_, O_3_, and NO_2_ (per 5 μg/m^3^ increase) on Hb concentration and the prevalence of anaemia. As we had observed strong correlations between the pollutants (Table S1 in the **Online Supplementary Document**), we sequentially developed each model separately for each pollutant, considering only one air pollutant each time. The classifications of the related variables below are detailed elsewhere (Table S2 in the **Online Supplementary Document**).

Model 1 included only a random intercept for the type of enterprise economy, accounting for its potential influence on occupational environments, management policies, or work cultures. Model 2 was additionally adjusted for the demographic characteristics, including gender and age. Model 3 further incorporated adjustment for occupational variables: enterprise size, length of service, industrial classification, and whether participants were exposed to anaemia-related occupational factors in their daily work [27,28]. Model 4 additionally adjusted for the annual average exposure to temperature and humidity.

Limited collinearity between pairwise variables was indicated when all variance inflation factors were less than 5. We incorporated an interaction term (stratified variable × air pollution) into the final model to examine the potential effects of fundamental features and occupational variables on the relationship between air pollutants and anaemia.

We subsequently performed stratified analyses by demographic characteristics and occupational factors in the final model to identify possible effect modifiers. Age and length of service were categorised into quartiles. The main model introduced an interaction term (stratification variable × air pollution) to evaluate the effects of interactions between each stratification variable and pollutant on worker anaemia.

Lastly, we performed sensitivity analyses to confirm the robustness of the estimated effects by excluding ‘indefinite’ enterprise types; including all participants with non-missing length of service (including those with less than 12 months of service); setting up multi-pollutant model analyses to more clearly disentangle the independent effects of each pollutant (Text S1 in the **Online Supplementary Document**).

We performed all analyses in *R*, version 4.2.3 (R Core Team, Vienna, Austria). A two-sided *P*-value of <0.05 indicated statistical significance for estimates.

## RESULTS

**Figure 1:**
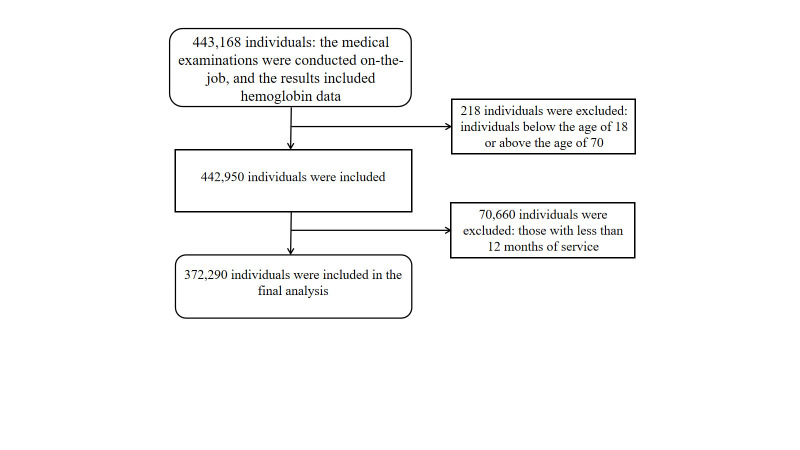
Inclusion and exclusion criteria and flowchart of the study population.

The initial data set included 443 168 individuals who underwent an on-the-job physical examination and whose physical examination results contained their Hb data. For our analysis, we removed any persons aged <18 years or >70 years and those with <1 year of service (n = 70 878)., leaving 372 290 individuals for our analysis (**Figure 1**).

There were 14 577 (3.9%) persons with anaemia in our sample (**Table 1**). Compared to those without anaemia, the individuals with the condition were older (40.40 years, standard deviation (SD) = 7.99 *vs*. 37.98 years, SD = 9.31), had a shorter period of service (64.34 months, SD = 61.07 *vs*. 73.00 months, SD = 69.61), were predominantly female (72.6% *vs*. 23.0%), and were more exposed to lead (6.8% *vs*. 4.7%) and benzene (36.2% *vs*. 28.6%) in their routine work. They were also more likely to work in small (42.1% *vs*. 39.7%) and private enterprises (45.2% *vs*. 41.7%), and be involved in the manufacturing sector (83.4% *vs*. 73.4%). Lastly, individuals with anaemia exhibited a marginally lower average annual temperature (23.61°C, SD = 0.53 *vs*. 23.66°C, SD = 0.52).

**Table 1:** Descriptive characteristics of 372 290 workers in this study*

Characteristics	Total (n = 372 290)	Non-anaemia (n = 357 713)	Anaemia (n = 14 577)	*P*-value
**Hb in μg/m^3^, x̄ (SD)**	146.02 (18.17)	148.06 (14.84)	95.87 (20.44)	<0.001
**Age in years, x̄ (SD)**	38.08 (9.28)	37.98 (9.31)	40.40 (7.99)	<0.001
**Age groups in years**				<0.001
18–31	87 775 (23.6)	85 989 (24.0)	1786 (12.3)	
32–38	98 228 (26.4)	94 912 (26.5)	3316 (22.7)	
39–46	92 718 (24.9)	87 593 (24.5)	5125 (35.2)	
>46	93 569 (25.1)	89 219 (24.9)	4350 (29.8)	
**Sex**				<0.001
Male	279 264 (75.0)	275 265 (77.0)	3999 (27.4)	
Female	93 026 (25.0)	82 448 (23.0)	10 578 (72.6)	
**Length of service in months, x̄ (SD)**	72.66 (69.32)	73.00 (69.61)	64.34 (61.07)	<0.001
**Length of service in months**				<0.001
12–23	88 124 (23.7)	84 108 (23.5)	4016 (27.6)	
24–47	90 874 (24.4)	87 251 (24.4)	3623 (24.9)	
48–95	99 431 (26.7)	95 595 (26.7)	3836 (26.3)	
>96	93 861 (25.2)	90 759 (25.4)	3102 (21.3)	
**Enterprise size**				<0.001
Small	148 147 (39.8)	142 003 (39.7)	6144 (42.1)	
Medium	104 090 (28.0)	99 772 (27.9)	4318 (29.6)	
Large	100 111 (26.9)	96 790 (27.1)	3321 (22.8)	
Indefinite	19 942 (5.4)	19 148 (5.4)	794 (5.4)	
**Business type**				<0.001
Enterprises from Hong Kong, Macao, and Taiwan	69 662 (18.7)	65 820 (18.4)	3842 (26.4)	
Individual economy	422 (0.1)	408 (0.1)	14 (0.1)	
Shareholding economy	3752 (1.0)	3524 (1.0)	228 (1.6)	
State-owned economy	47 172 (12.7)	46 440 (13.0)	732 (5.0)	
Collective economy	1221 (0.3)	1186 (0.3)	35 (0.2)	
Associate economy	2533 (0.7)	2440 (0.7)	93 (0.6)	
Private economy	155 738 (41.8)	149 145 (41.7)	6593 (45.2)	
Foreign investment	51 570 (13.9)	49 815 (13.9)	1755 (12.0)	
Other	40 220 (10.8)	38 935 (10.9)	1285 (8.8)	
**Industrial classification**				<0.001
Manufacturing	274 612 (73.8)	262 449 (73.4)	12 163 (83.4)	
Production and supply of electricity, heat, gas, and water	16 973 (4.6)	16 728 (4.7)	245 (1.7	
Construction industry	12 567 (3.4)	12 218 (3.4)	349 (2.4)	
Transportation, warehousing, and postal industry	17 592 (4.7)	17 414 (4.9)	178 (1.2)	
Wholesale and retail trade	23 567 (6.3)	22 683 (6.3)	884 (6.1)	
Other	26 979 (7.2)	26 221 (7.3)	758 (5.2)	
**Exposure to lead during routine work**				<0.001
Yes	17 674 (4.7)	16 678 (4.7)	996 (6.8)	
No	354 616 (95.3)	341 035 (95.3)	13 581 (93.2)	
**Exposure to benzene during routine work**				
Yes	107 581 (28.9)	102 308 (28.6)	5273 (36.2)	
No	264 709 (71.1)	255 405 (71.4)	9304 (63.8)	
**Humidity, x̄ (SD)**	74.44 (3.28)	74.44 (3.29)	74.45 (3.21)	0.803
**Temperature, x̄ (SD)**	23.66 (0.52)	23.66 (0.52)	23.61 (0.53)	<0.001

Hb – haemoglobin, SD – standard deviation, x̄ – mean

*Values are presented as n (%) unless specified otherwise.

### Association of exposure to five air pollutants with Hb concentration and the prevalence of anaemia

Exposure to PM_2.5_, PM_10_, PM_coarse_, O_3_, and NO_2_ was significantly correlated with lower Hb concentration (**Table 2**). An increase of 5 μg/m^3^ in PM_2.5_, PM_10_, PM_coarse_, O_3_, and NO_2_ concentrations corresponded to a decrease in Hb concentration (β) by 2.037 g/L (95% confidence interval (CI) = 1.938, 2.137), 1.096 g/L (95% CI = 1.040, 1.152), 1.412 g/L (95% CI = 1.313, 1.510), 0.518 g/L (95% CI = 0.489, 0.547), and 0.250 g/L (95% CI = 0.219, 0.281), respectively.

**Table 2:** Associations between ambient air pollution exposure and haemoglobin levels, β (95% CI)

	PM_2.5_	PM_10_	PM_coarse_	O_3_	NO_2_
**Model 1**	−2.551 (−2.672, −2.431)	−0.893 (−0.958, −0.827)	−0.418 (−0.530, −0.307)	−0.221 (−0.256, −0.186)	−0.095 (−0.132, −0.057)
**Model 2 (model 1 + basic characteristics)**	−2.109 (−2.204, −2.013)	−0.955 (−1.007, −0.904)	−0.983 (−1.072, −0.894)	−0.458 (−0.486, −0.430)	−0.251 (−0.281, −0.221)
**Model 3 (model 2 + occupational variables)**	−2.014 (−2.111, −1.917)	−0.946 (−0.999, −0.894)	−1.025 (−1.116, −0.935)	−0.469 (−0.497, −0.441)	−0.248 (−0.278, −0.218)
**Model 4 (model 3+ meteorological variables)**	−2.037 (−2.137, −1.938)	−1.096 (−1.152, −1.040)	−1.412 (−1.510, −1.313)	−0.518 (−0.547, −0.489)	−0.250 (−0.281, −0.219)

β – regression coefficients, CI – confidence interval, NO_2_ – nitrogen dioxide, O_3_ – ozone, PM10 – particulate matter ≤10 μm in aerodynamic diameter, PM_2.5_ – particulate matter ≤2.5 μm in aerodynamic diameter, PM_coarse_ – particulate matter 2.5–10 μm in aerodynamic diameter

Exposure to PM_2.5_, PM_10_, and PM_coarse_ was associated with a higher prevalence of anaemia (**Table 3**). The prevalence of anaemia increased by 11.3% (95% CI = 7.3%, 15.5%) for every 5 μg/m^3^ increase in PM_2.5_ concentration. Similarly, the OR estimates for anaemia increased by 5.0% (95% CI = 2.8%, 7.3%) and 4.5% (95% CI = 0.6%, 8.5%) for a 5 μg/m^3^ increase in PM_10_ and PM_coarse_, respectively. However, no significant association was observed between elevated O_3_ and NO_2_ concentrations and the prevalence of anaemia.

**Table 3:** Associations between ambient air pollution exposure and the prevalence of anemia, OR (95% CI)

	PM_2.5_	PM_10_	PM_coarse_	O_3_	NO_2_
**Model 1**	1.180 (1.140, 1.222)	1.033 (1.014, 1.053)	0.949 (0.918, 0.980)	0.975 (0.965, 0.984)	0.980 (0.969, 0.991)
**Model 2 (model 1 + basic characteristics)**	1.126 (1.087, 1.167)	1.039 (1.018, 1.059)	1.002 (0.969, 1.037)	0.995 (0.985, 1.005)	0.996 (0.985, 1.007)
**Model 3 (model 2 + occupational variables)**	1.104 (1.065, 1.144)	1.032 (1.011, 1.053)	1.002 (0.968, 1.037)	0.997 (0.987, 1.008)	0.993 (0.982, 1.005)
**Model 4 (model 3 + meteorological variables)**	1.113 (1.073, 1.155)	1.050 (1.028, 1.073)	1.045 (1.006, 1.085)	1.003 (0.992, 1.014)	0.998 (0.986, 1.010)

CI – confidence interval, NO_2_ – nitrogen dioxide, O_3_ – ozone, OR – odds ratio, PM10 – particulate matter ≤10 μm in aerodynamic diameter, PM_2.5_ – particulate matter ≤2.5 μm in aerodynamic diameter, PM_coarse_ – particulate matter 2.5–10 μm in aerodynamic diameter

### Potential effect modifiers underlying the association between anaemia and air pollutant exposures

Workers aged 18–31 years were the most susceptible to the effects of PM_2.5_ (odds ratio (OR) = 1.181; 95% CI = 1.066, 1.308) and PM_10_ (OR = 1.067; 95% CI = 1.006, 1.131) on anaemia compared to other age groups (Table S3 in the **Online Supplementary Document**). The impact of PM_coarse_ (OR = 1.072; 95% CI = 1.001, 1.149) was greatest in workers over 46 years of age. Although the effects of O_3_ and NO_2_ on anaemia varied across age groups, they were not significant, as the CIs for OR values crossed the threshold of no effect. Age may also amplify the association between exposure to PM_2.5_, PM_10_, and PM_coarse_, and anaemia (*P <* 0.01). Furthermore, we noted differences between genders regarding the effects of various air pollutant exposures on the prevalence of anaemia. Males were more affected by PM_2.5_ (OR = 1.271; 95% CI = 1.188, 1.360) and PM_10_ (OR = 1.075; 95% CI = 1.035, 1.116), while females were more susceptible to the effects of PM_coarse_ (OR = 1.077; 95% CI = 1.028, 1.128) and O_3_ (OR = 1.021; 95% CI = 1.008, 1.034).

The effects of PM_2.5_ (OR = 1.167) and PM_10_ (OR = 1.055) were the greatest among those with 24–27 and 12–23 months of service, respectively. However, these differences were not statistically significant. Workers with 12–23 months of service were more sensitive to the effect of NO_2_ (OR = 1.041), and those with 48–96 months of service were more sensitive to PM_coarse_ (OR = 1.080) compared to other groups. As for enterprise size, we found that the OR estimates were higher in large (PM_2.5_: OR = 1.308) and indefinite enterprises (PM_10_: OR = 1.214; PM_coarse_: OR = 1.827; NO_2_: OR = 1.128; O_3_: OR = 1.326). We further noted significant modification effects for different industrial classifications, where participants working in the manufacturing sector were most affected by PM_10_ and PM_coarse_. While the effects of PM_2.5_ and O_3_ on anaemia were larger in other industrial groups, they were statistically significant in the manufacturing subgroup only.

We also studied the effects of lead and benzene. Among these five air pollutants, *i.e.* PM_2.5_, PM_10_, PM_coarse_, O_3_, and NO_2_, and NO₂., we did not observe significant interaction effects of co-exposure, except for O_3_ and lead (*P <* 0.01). Although we found no significant differences for NO_2_ and O_3,_ we did see significant associations for other pollutants in non-benzene exposed population (PM_2.5_: OR = 1.137; 95% CI = 1.086, 1.190; PM_10_: OR = 1.064; 95% CI = 1.038, 1.092; PM_coarse_: OR = 1.076; 95% CI = 1.027, 1.127).

### Sensitivity analysis

We obtained similar findings to our main analyses when excluding participants from enterprises with ‘indefinite’ types, when including participants with all lengths of service, and when using multi-pollutant, as opposed to single-pollutant models (Tables S4–6 in the **Online Supplementary Document**).

## DISCUSSION

We found that exposure to PM_2.5_, PM_10_, PM_coarse_, O_3_, and NO_2_ was significantly associated with decreased Hb concentration and with increased odds of anaemia, albeit with variations across demographic characteristics and occupational factors. In particular, we found that lead exposure may enhance the effect of ozone exposure on the prevalence of anaemia. Benzene exposure, meanwhile, may weaken the anemic impact of air pollutants.

### Effects of five conventional air pollutants on Hb and anaemia

Some evidence exists regarding the association of the five pollutants with Hb levels and the prevalence of anemia. In a cross-sectional study of elderly individuals in China, each interquartile range increase in PM_10_ and PM_2.5_ concentrations corresponded to a 5% (95% CI = 2–9) and 11% (95% CI = 6–16) increase in anaemia prevalence, respectively, while, for each IQR increment in PM_1_ and NO_2_, Hb concentrations decreased by 0.55 (95% CI = 0.41, 0.69) and 1.71 (95% CI = 1.57, 1.85) mg/dL [8]. Similar findings were reported in other areas such as the USA [9] and Peru [11]. While our findings on particulate pollutants (PM, including PM_2.5_, PM_10_, and PM_coarse_) closely align with those of previous studies, we did not observe a statistically significant increase in the prevalence of anaemia with NO_2_, and could not draw robust conclusions from our results on O_3_. These discrepancies may stem from differences in geographical regions, exposure durations, and study populations. Previous studies primarily focussed on elderly individuals, pregnant women, and children, whereas our research concentrated on workers. Such different exposure concentrations and population susceptibilities may have contributed to the observed differences. In our study, the effects of NO_2_ and O_3_ on Hb may be less pronounced compared to PM, with their impact related to a decrease in Hb concentration, but not with an increase in anaemia prevalence. Furthermore, the ‘healthy worker effect’ may also explain the lack of association for NO_2_ and O_3_ exposure in our study [29].

The associations we observed are consistent with established mechanistic pathways. Systemic inflammation is a key factor underlying the risk of anaemia, with research showing that PM_2.5_ exposure can induce systemic inflammation, affecting anaemia-related blood cell parameters [30,31]. Long-term exposure to NO_2_ and SO_2_ also correlates with increased inflammatory markers [32]. In animal models, PM_2.5_ exposure harms the bone marrow microenvironment, particularly in young mice [33,34]. Air pollutants can alter iron homeostasis via direct cellular mechanisms, such as complexing with or displacing iron, which leads to a functional deficiency and subsequent inflammation [35]. PM_2.5_ is known to directly impair erythropoiesis by significantly down-regulating the expression of erythropoietin, ultimately suppressing blood cell formation [36]. Hemolysis or erythrocyte destruction, which may be brought on by mineral particles adsorbed following exposure to air pollution, is another explanation for the connection between air pollution and anaemia [37].

### Potential modifiers of the association between air pollution and Hb and anaemia

We observed discrepancies in the effects of different air pollutant exposures on the prevalence of anaemia among men and women. The former were more susceptible to anaemia due to PM_2.5_ and PM_10_ exposure, where the latter were more affected by PM_coarse_ and O_3_. These inconsistencies may be partly explained by differences in occupational types between the two genders, resulting in varying pollutant exposure levels [38]. Other confounding factors, such as higher probability of outdoor exposure among men and increased exposure to cooking fumes among women, could also explain the observed effects [39,40]. Differences in length of service and age may also be associated with variations in exposure to different pollutants. These factors, however, warrant further analyses and confirmations.

The higher vulnerability among the participants engaged in manufacturing was expected. According to a study about the exposure of PM_2.5_ pollution and inequality in the Chinese population, public government employees and tertiary industry service personnel had a lower risk of exposure to PM_2.5_ concentration compared with long-term engaged manufacturing workers and professional and technical workers, due to the welcoming office environment and cutting-edge air purification equipment [41]. Notably, there were higher OR estimates in large and indefinite enterprises. Although no research directly investigated the role that enterprise size plays in the impact of air pollution on anaemia, a study suggested that air pollution had a greater impact on workers in large enterprises in terms of obstructive ventilatory dysfunction [42], potentially due to differing levels of exposure to workplace air pollutants [43]. An analysis in Tianjin found similar results, showing that the majority of occupational disease cases (such as pneumoconiosis and otolaryngological disorders) occurred in state-owned large and medium-sized enterprises [44]. In addition, the basic characteristics of participants in different enterprises and the lower proportion of indefinite enterprise participants may also be the reasons for the interesting results. This analysis suggests a potential need for increased attention to the health of workers in large enterprises. Regulatory authorities may consider strengthening guidance for key companies, particularly large enterprises, to improve working conditions and ensure timely occupational health screenings for employees. It is important to note that the findings from our stratified analyses are exploratory and warrant further verification through future studies with more rigorous statistical methodologies.

### Effects of anaemia-related occupational factors on the association of air pollution with Hb levels and anaemia prevalence

Regarding the impact of anaemia-related occupational variables (such as lead and benzene exposure) on the air pollution-anaemia association, our results indicate that among individuals exposed to lead, exposure to PM_10_, PM_coarse_, NO_2_, and O_3_ may be associated with higher odds of anaemia. However, we observed that the effects of air pollutants on anaemia were more pronounced in the non-benzene exposure population, for which we proposed several possible hypotheses.

First, this may be attributed to a masking effect, where one study reported that male workers who were chronically exposed to gasoline and air pollution exhibited a compensatory increase in Hb and red blood cells [45]. The increase in Hb might have masked the effect of air pollution on Hb and anaemia.

Second, workers chronically exposed to benzene may have already developed adaptive physiological responses. A previous study has shown that occupational benzene exposure can significantly increase reactive oxygen species levels and induce the expression of the tumor suppressor gene p53 [46]. This suggests that long-term exposure to benzene may cause the body to continuously activate stress defense pathways to maintain homeostasis. Such adaptive physiological mechanisms may, to some extent, alter the body’s sensitivity to other environmental toxicants, such as air pollutants.

Third, the ‘healthy worker effect’ must be considered, whereby workers suffering from severe anaemia may leave the workforce, biasing the results toward the null. A prospective study involving 17 566 individuals from the general population showed that combined exposure to multiple air pollutants, including PM_2_._5_, NO_2_, and O_3_, was associated with anaemia. Both the multi-pollutant model (OR = 1.29) and the single-pollutant models (OR>1.20) yielded effect estimates consistently higher than the association level observed in our study’s occupationally benzene-exposed population (OR = 1.137) [47]. Given that occupational populations typically have better baseline health status, the healthy worker effect may have led to an underestimation of the true effect in our study. However, as we used a cross-sectional design, we were unable to collect information on employment history, job transfers, and attrition, making it impossible to quantify this impact.

Furthermore, the possibility of differential measurement error among benzene-exposed individuals cannot be excluded. Specifically, the binary exposure information (*i.e.* whether a worker was exposed to benzene) was provided by employers based on the presence of hazardous substances at different job positions, rather than being derived from direct measurements of ambient benzene concentrations. This approach may have introduced a certain degree of differential measurement error.

As for interaction effects, we noted a significant interaction between lead and O_3_ exposures. Previous research has explored the potential mechanisms of occupational variables associated with anaemia. For example, lead exposure has been shown to impede the activity of pyrimidine 5′-nucleotidase, diminish heme content, and trigger phagocytosis and externalisation of phosphatidylserine in red blood cells [48]. It could also increase the production of reactive oxygen species, potentially leading to damage of erythrocyte membranes and the occurrence of haemolytic anaemia [49]. Iron-dependent ferroptosis could also be involved in benzene-induced inflammatory anaemia [27]. However, we did not observe a statistically significant interaction between other pollutants and lead or benzene exposure, indicating that further investigations should test these hypotheses.

### Strengths and limitations

To the best of our knowledge, no prior epidemiological research has concurrently examined the effects of long-term ambient air pollution exposure on both Hb concentrations and anaemia among Chinese workers. Our study addresses this gap based on a large sample size of over 300 000 individuals. Nonetheless, we acknowledge some limitations. First, due to data limitations, we were unable to further explore occupational-specific mechanisms (*e.g.* combined metal-pollutant toxicity), establish a non-worker control group to facilitate more occupational-specific interpretations, or stratify results by anaemia subtypes to achieve a better clinical understandings.Although we adjusted for several important occupational factors, we could not address residual confounding from other occupational factors such as noise, shift work, and ergonomic stress. Second, we utilised modeled exposure assessments, rather than personal monitoring. Although this approach is commonly used for exposure evaluation, including in studies that utilised the CHAP database [50–52], it is susceptible to exposure misclassification, as individual-level variability in exposure cannot be captured. Third, given the potential influence of the healthy worker effect, the generalisability of our findings may be limited, particularly when extrapolated to vulnerable populations such as older adults and individuals with chronic conditions. Lastly, we conducted exploratory subgroup analyses without formal correction for multiple comparisons. Therefore, these findings should be interpreted with caution.

## CONCLUSIONS

In our sample, ambient air pollution exposure was associated with decreased Hb concentrations and increased anaemia prevalence in workers. Demographic characteristics (age and gender) and occupational factors (length of service, industrial classification, enterprise size, and exposure to anaemia-related variables) could influence this relationship. These findings could support the development of preventive programmes against anaemia that would target environmental and occupational factors.

## Additional material


Online Supplementary Document
