# Supplementary material for: Ambient air pollutants, increased anaemia risk, and vulnerable subgroups: evidence from a large group of workers in South China
Source: J Glob Health. 2025 Dec 19;15:04346. doi: 10.7189/jogh.15.04346 (PMC12715747; doi:10.7189/jogh.15.04346)
Supplement: Online Supplementary Document [file jogh-15-04346-s001.pdf]

Supplement to: Li X, Wu Z, Zhao Y, Chen X, Li Z, Sun Y, Gong Y, Hu P, Huang X, Pan W, Xie S, Zhang W, Huang Y. Ambient air pollutants, increased anaemia risk, and the vulnerable subgroups: evidence from a large group of workers in South China. J Glob Health. 2025;15:04346.

Included Files

Supplementary Text 1. Multi-pollutant model supplemental analysis.

Table S1. Pollutant descriptive statistics and correlation matrix.

Table S2. Classifications of the related variables in the models.

Table S3. Subgroup analyses for the association between air pollution and the prevalence of anemia.

Table S4. Sensitivity analysis.

Table S5. Association between air pollutants and hemoglobin levels in multi-pollutant and single-pollutant models.

Table S6. Association between air pollutants and anemia prevalence in multi-pollutant and single-pollutant models.

STROBE Statement--Checklist of items that should be included in reports of *cross-sectional studies*

The report according to the GRABDROP guidelines of JoGH

Supplementary Text 1. Multi-pollutant model supplemental analysis.

We developed multi-pollutant models to more clearly elucidate the independent effect of each pollutant. Previous studies have demonstrated that both particulate matter and gaseous pollutants exert adverse effects on human health. Among the combined effects of gaseous pollutants on excess mortality risk, NO_2 has been identified as the most prominent contributor. However, under exposure scenarios where NO_2 coexists with particulate matter, $PM_{2.5}$ often predominates in driving mortality effects, suggesting that the harmful impact of NO_2 may be largely masked by particulate matter, particularly $PM_{2.5}$ [1]. Based on this evidence, we analyzed particulate and gaseous pollutants separately to better clarify their independent associations with health risks. In the particulate matter model, considering that PM_{10} consists of both $PM_{2.5}$ and PM_{coarse}, inclusion of PM_{10} would lead to multicollinearity; therefore, $PM_{2.5}$ and PM_{coarse} instead of PM_{10} were included in the model. In the gaseous pollutant model, NO_2 and O_3 were included. We agree with the Reviewer that multi-pollutant models may be helpful; however, we may not include too many highly-correlated variables in the same model, because from a statistical perspective, such models can be very unstable in estimation. Nevertheless, we respect the Reviewer's comments and have therefore added some results from multi-pollutant models.

As shown in **Table S5**, in multi-pollutant models, $PM_{2.5}$, PM_{coarse}, and O_3 were all associated with decreases in hemoglobin levels, and these associations were statistically significant, consistent with the trends observed in the single-pollutant models. Although NO_2 appeared to be associated with increased hemoglobin levels in the multi-pollutant model, we speculate that this may be due to estimation bias caused by multicollinearity. In atmospheric chemical processes, NO_2 can be converted into O_3 through a series of photochemical reactions, and anthropogenic emissions can accelerate this transformation [2]. Therefore, NO_2 and O_3 are often highly correlated in the environment. When both pollutants are included in the same statistical model, multicollinearity may lead to biased effect estimates.

In terms of the prevalence of anemia, both the concentrations of $PM_{2.5}$ and O_3

displayed a positive association with the prevalence, as shown in **Table S6**. It is also consistent with the effects observed in the single-pollutant models. Although the odds ratio (OR) for PM_{coarse} was less than 1 in the multi-pollutant model, the association was not statistically significant. The findings for NO_2 were also consistent between the multi-pollutant and single-pollutant models.

Supplementary table

Table S1. Pollutant descriptive statistics and correlation matrix.

	Mean±SD (μg/m^3)	Min (μg/m^3)	Max (μg/m^3)	IQR (μg/m^3)	Pearson correlation coefficients				
					$PM_{2.5}$	PM_{10}	PM_{coarse}	O_3	NO_2
$PM_{2.5}$	24.52± 2.43	14.74	33.71	3.47	1	0.88	0.59	0.72	0.59
PM_{10}	43.63± 4.52	29.47	58.57	6.88		1	0.9	0.77	0.74
PM_{coarse}	19.11± 2.64	10.65	29.18	3.81			1	0.66	0.73
O_3	103.72± 8.37	79.17	130.71	12.90				1	0.6
NO_2	29.61± 7.94	10.67	57.21	11.49					1

Abbreviations: SD: standard deviation; $PM_{2.5}$: particulate matter ≤ 2.5μm in aerodynamic diameter; PM_{10}: particulate matter ≤ 10μm in aerodynamic diameter; PM_{coarse}: particulate matter 2.5-10 μm in aerodynamic diameter; O_3: ozone; NO_2: nitrogen dioxide.

Table S2. Classifications of the related variables in the models.

Variables	Classifications
Type of enterprise economy	State-owned economy; Collective economy; Associate economy; Private economy; Individual economy; Shareholding economy; Hong Kong, Macao, and Taiwan economy; Foreign investment; Others
Gender	Male; Female
Age	18-31 years old; 32-38 years old; 39-46 years old; > 46 years old
Length of service (the continuous working time of workers in the enterprise)	12-23 months; 24-47 months; 48-95 months; > 96 months
Enterprise size[a]	Small; Medium; Large; Indefinite
Industrial classification[b]	Manufacturing; production and supply of electricity, heat, gas, and water; Construction industry; Transportation, warehousing, postal industry; Wholesale and retail trade; Others
Anemia-related occupational variables	Lead; Benzene

[a]Classification criteria was based on the ‘Statistical Division of Large, Medium and Small Micro Enterprises (2017) (No. 213)’ issued by the National Bureau of Statistics in 2017

[b]classification criteria based on the National Economic Industry Classification (GB/T 4754-2017), with industries accounting for less than 1% classified as ‘Others’.

Table S3. Subgroup analyses for the association between air pollution and the prevalence of anemia.

	$PM_{2.5}$		PM_{10}		PM_{coarse}		O_3		NO_2	
	OR (95% *CI*)	$P_{for\ interaction}$	*OR* (95% *CI*)	$P_{for\ interaction}$	*OR* (95% *CI*)	$P_{for\ interaction}$	*OR* (95% *CI*)	$P_{for\ interaction}$	*OR* (95% *CI*)	$P_{for\ interaction}$
**Length of service groups**		**0.936**		**0.345**		**<0.01**		**<0.01**		**<0.01**
12-23	1.125 (1.047, 1.209)		1.055 (1.012, 1.100)		1.045 (0.970, 1.127)		0.981 (0.960, 1.003)		1.041 (1.017, 1.065)	
24-47	1.167 (1.084, 1.256)		1.050 (1.006, 1.096)		0.986 (0.912, 1.067)		1.004 (0.982, 1.026)		0.971 (0.948, 0.996)	
48-96	1.084 (1.010, 1.164)		1.052 (1.010, 1.096)		1.080 (1.004, 1.162)		1.020 (0.998, 1.041)		0.985 (0.962, 1.009)	
＞96	1.066 (0.983, 1.155)		1.046 (0.999, 1.094)		1.080 (0.997, 1.170)		1.016 (0.994, 1.039)		1.012 (0.986, 1.038)	
**Age groups**		**<0.01**		**<0.01**		**<0.01**		**<0.01**		**<0.01**
18-31	1.181 (1.066, 1.308)		1.067 (1.006, 1.131)		1.033 (0.927, 1.150)		0.970 (0.941, 0.999)		1.018 (0.985, 1.052)	
32-38	1.146 (1.062, 1.236)		1.055 (1.010, 1.102)		1.028 (0.949, 1.113)		1.008 (0.986, 1.031)		0.993 (0.969, 1.018)	
39-46	1.082 (1.016, 1.153)		1.036 (0.999, 1.074)		1.030 (0.965, 1.099)		1.006 (0.988, 1.025)		0.996 (0.976, 1.017)	
＞46	1.107 (1.035, 1.185)		1.057 (1.017, 1.099)		1.072 (1.001, 1.149)		1.015 (0.995, 1.035)		0.997 (0.976, 1.019)	
**Sex**		**<0.01**		**0.079**		**<0.01**		**<0.01**		**<0.01**
Male	1.271 (1.188, 1.360)		1.075 (1.035, 1.116)		0.992 (0.928, 1.060)		0.958 (0.939, 0.978)		0.987 (0.966, 1.008)	
Female	1.062 (1.017, 1.109)		1.044 (1.018, 1.071)		1.077 (1.028, 1.128)		1.021 (1.008, 1.034)		1.005 (0.991, 1.020)	

**Enterprise size**		**<0.01**		**<0.01**		**0.027**		**<0.01**		**<0.01**
Small	1.044 (0.989, 1.103)		1.023 (0.991, 1.056)		1.028 (0.971, 1.088)		0.988 (0.971, 1.004)		0.973 (0.955, 0.991)	
Medium	1.168 (1.093, 1.249)		1.071 (1.030, 1.113)		1.052 (0.978, 1.133)		1.011 (0.991, 1.030)		1.014 (0.991, 1.037)	
Large	1.308 (1.196, 1.431)		1.101 (1.049, 1.156)		1.047 (0.964, 1.137)		1.027 (1.003, 1.052)		0.997 (0.970, 1.024)	
Indefinite	0.996 (0.828, 1.198)		1.214 (1.095, 1.346)		1.827 (1.517, 2.200)		1.128 (1.069, 1.190)		1.326 (1.252, 1.403)	
**Industrial classsifications**		**<0.01**		**0.020**		**<0.01**		**<0.01**		**<0.01**
Manufacturing	1.120 (1.077, 1.166)		1.062 (1.037, 1.087)		1.074 (1.028, 1.121)		1.015 (1.003, 1.027)		1.010 (0.996, 1.024)	
Production and supply of electricity, heat, gas and water	1.546 (1.051, 2.272)		1.179 (0.969, 1.436)		1.130 (0.833, 1.533)		0.968 (0.870, 1.076)		1.119 (1.010, 1.239)	
Construction industry	1.077 (0.832, 1.395)		1.029 (0.886, 1.195)		1.012 (0.773, 1.326)		1.024 (0.952, 1.101)		1.005 (0.924, 1.092)	
Transportation, warehousing and postal industry	1.356 (0.955, 1.925)		1.173 (0.965, 1.425)		1.250 (0.869, 1.797)		1.131 (1.012, 1.265)		1.081 (0.983, 1.190)	
Wholesale and retail trade	0.835 (0.710, 0.982)		0.963 (0.884, 1.049)		1.034 (0.900, 1.190)		0.925 (0.884, 0.967)		0.996 (0.957, 1.037)	

Other	1.560 (1.314, 1.853)		1.071 (0.976, 1.175)		0.827 (0.706, 0.970)		0.953 (0.910, 0.997)		0.890 (0.850, 0.932)	
**Lead**		**0.289**		**0.069**		**0.054**		**<0.01**		**0.198**
Yes	1.094 (0.951, 1.258)		1.080 (0.989, 1.179)		1.173 (0.987, 1.394)		1.023 (0.983, 1.065)		1.019 (0.968, 1.073)	
No	1.107 (1.065, 1.151)		1.046 (1.023, 1.069)		1.037 (0.997, 1.078)		0.998 (0.987, 1.009)		0.996 (0.984, 1.008)	
**Benzene**		**0.071**		**0.131**		**0.194**		**0.919**		**0.149**
Yes	1.085 (1.020, 1.155)		1.024 (0.987, 1.063)		0.981 (0.917, 1.049)		1.000 (0.981, 1.019)		0.985 (0.964, 1.006)	
No	1.137 (1.086, 1.190)		1.064 (1.038, 1.092)		1.076 (1.027, 1.127)		1.004 (0.990, 1.017)		1.004 (0.990, 1.019)	

Abbreviations: *OR*: odds ratio; *CI*: confidence interval; *P*: *P* for interaction; $PM_{2.5}$: particulate matter ≤ 2.5μm in aerodynamic diameter; PM_{10}: particulate matter ≤10μm in aerodynamic diameter; PM_{coarse}: particulate matter 2.5-10μm in aerodynamic diameter; NO_2: nitrogen dioxide; O_3: ozone

Table S4. Sensitivity analysis.

	Databases without unknown enterprise size	Database including all working ages
Hb	*β* (95% *CI*)	*β* (95% *CI*)
$PM_{2.5}$	-2.100 (-2.199, -2.001)	-1.967 (-2.059, -1.876)
PM_{10}	-1.116 (-1.171, -1.060)	-1.065 (-1.117, -1.014)
PM_{coarse}	-1.410 (-1.509, -1.310)	-1.375 (-1.466, -1.283)
O_3	-0.529 (-0.558, -0.500)	-0.486 (-0.513, -0.460)
NO_2	-0.211 (-0.242, -0.179)	-0.232 (-0.261, -0.204)
Anemia	*OR* (95% *CI*)	*OR* (95% *CI*)
$PM_{2.5}$	1.127 (1.085, 1.170)	1.111 (1.074, 1.150)
PM_{10}	1.051 (1.028, 1.074)	1.044 (1.024, 1.065)
PM_{coarse}	1.031 (0.992, 1.073)	1.027 (0.992, 1.064)
O_3	1.003 (0.992, 1.014)	0.999 (0.989, 1.009)
NO_2	0.985 (0.973, 0.997)	0.996 (0.985, 1.007)

Abbreviations: Hb: hemoglobin; *β:* regression coefficients; *CI*: confidence interval; *OR*: odds ratio; $PM_{2.5}$: particulate matter ≤ 2.5μm in aerodynamic diameter; PM_{10}: particulate matter ≤ 10μm in aerodynamic diameter; PM_{coarse}: particulate matter 2.5-10 μm in aerodynamic diameter; O_3: ozone; NO_2: nitrogen dioxide.

Table S5. Association between air pollutants and hemoglobin levels in multi-pollutant and single-pollutant models.

Air pollutants	*β (95% CI)*
Single pollutant	
$PM_{2.5}$	-2.037 (-2.137, -1.938)
PM_{10}	-1.096 (-1.152, -1.040)
PM_{coarse}	-1.412 (-1.510, -1.313)
O_3	-0.518 (-0.547, -0.489)
NO_2	-0.250 (-0.281, -0.219)
Multiple pollutants	
$PM_{2.5}$	-1.814 (-1.935, -1.693)
PM_{coarse}	-0.388 (-0.508, -0.268)
O_3	-0.568 (-0.603, -0.533)
NO_2	0.095 (0.057, 0.133)

Abbreviations: *β:* regression coefficients; *CI*: confidence interval; $PM_{2.5}$: particulate matter ≤ 2.5μm in aerodynamic diameter; PM_{10}: particulate matter ≤ 10μm in aerodynamic diameter; PM_{coarse}: particulate matter 2.5-10 μm in aerodynamic diameter; O_3: ozone; NO_2: nitrogen dioxide.

Table S6. Association between air pollutants and anemia prevalence in multi-pollutant and single-pollutant models.

Air pollutants	*OR* (95% *CI*)
Single pollutant	
$PM_{2.5}$	1.113 (1.073, 1.155)
PM_{10}	1.050 (1.028, 1.073)
PM_{coarse}	1.045 (1.006, 1.085)
O_3	1.003 (0.992, 1.014)
NO_2	0.998 (0.986, 1.010)
Multiple pollutants	
$PM_{2.5}$	1.130 (1.081, 1.181)
PM_{coarse}	0.973 (0.929, 1.019)
O_3	1.006 (0.993, 1.019)
NO_2	0.994 (0.980, 1.009)

Abbreviations: *CI*: confidence interval; *OR*: odds ratio; $PM_{2.5}$: particulate matter ≤ 2.5μm in aerodynamic diameter; PM_{10}: particulate matter ≤ 10μm in aerodynamic diameter; PM_{coarse}: particulate matter 2.5-10 μm in aerodynamic diameter; O_3: ozone; NO_2: nitrogen dioxide.

References:

1. Yu Y, Tang Z, Huang Y, et al. Assessing long-term effects of gaseous air pollution exposure on mortality in the United States using a variant of difference-in-differences analysis. *Sci Rep*. 2024;14(1):16220. Published 2024 Jul 13. doi:10.1038/s41598-024-66951-9
2. Wang F, Wang W, Wang Z, et al. Drivers of PM2.5-O3 co-pollution: from the perspective of reactive nitrogen conversion pathways in atmospheric nitrogen cycling. Sci Bull (Beijing). 2022;67(18):1833-1836. doi: 10.1016/j.scib.2022.08.016

STROBE Statement—Checklist of items that should be included in reports of ***cross-sectional studies***

	Item No	Recommendation	Page No
**Title and abstract**	1	(*a*) Indicate the study's design with a commonly used term in the title or the abstract	1-4
		(*b*) Provide in the abstract an informative and balanced summary of what was done and what was found	3
**Introduction**			
Background/rationale	2	Explain the scientific background and rationale for the investigation being reported	5-6
Objectives	3	State specific objectives, including any prespecified hypotheses	6
**Methods**			
Study design	4	Present key elements of study design early in the paper	6-7
Setting	5	Describe the setting, locations, and relevant dates, including periods of recruitment, exposure, follow-up, and data collection	6-8
Participants	6	(*a*) Give the eligibility criteria, and the sources and methods of selection of participants	6-7
Variables	7	Clearly define all outcomes, exposures, predictors, potential confounders, and effect modifiers. Give diagnostic criteria, if applicable	9
Data sources/ measurement	8*	For each variable of interest, give sources of data and details of methods of assessment (measurement). Describe comparability of assessment methods if there is more than one group	*6-8*
Bias	9	Describe any efforts to address potential sources of bias	9-10
Study size	10	Explain how the study size was arrived at	7
Quantitative variables	11	Explain how quantitative variables were handled in the analyses. If applicable, describe which groupings were chosen and why	7-9
Statistical methods	12	(*a*) Describe all statistical methods, including those used to control for confounding	8-9
		(*b*) Describe any methods used to examine subgroups and interactions	9
		(*c*) Explain how missing data were addressed	NA
		(*d*) If applicable, describe analytical methods taking account of sampling strategy	9
		(*e*) Describe any sensitivity analyses	9
**Results**			
Participants	13*	(a) Report numbers of individuals at each stage of study—eg numbers potentially eligible, examined for eligibility, confirmed eligible, included in the study, completing follow-up, and analysed	NA
		(b) Give reasons for non-participation at each stage	NA
		(c) Consider use of a flow diagram	29
Descriptive data	14*	(a) Give characteristics of study participants (eg demographic, clinical, social) and information on exposures and potential confounders	10
		(b) Indicate number of participants with missing data for each variable of interest	NA
Outcome data	15*	Report numbers of outcome events or summary measures	10
Main results	16	(*a*) Give unadjusted estimates and, if applicable, confounder-adjusted estimates and their precision (eg, 95% confidence interval). Make clear which confounders were adjusted for and why they were included	10-12; 27-28
		(*b*) Report category boundaries when continuous variables were categorized	7
		(*c*) If relevant, consider translating estimates of relative risk into absolute risk for a meaningful time period	NA
Other analyses	17	Report other analyses done—eg analyses of subgroups and interactions, and sensitivity analyses	11-12
**Discussion**			
Key results	18	Summarise key results with reference to study objectives	12

Limitations	19	Discuss limitations of the study, taking into account sources of potential bias or imprecision. Discuss both direction and magnitude of any potential bias	17-18
Interpretation	20	Give a cautious overall interpretation of results considering objectives, limitations, multiplicity of analyses, results from similar studies, and other relevant evidence	13-17
Generalisability	21	Discuss the generalisability (external validity) of the study results	17
**Other information**			
Funding	22	Give the source of funding and the role of the funders for the present study and, if applicable, for the original study on which the present article is based	20

*Give information separately for exposed and unexposed groups.

Note: An Explanation and Elaboration article discusses each checklist item and gives methodological background and published examples of transparent reporting. The STROBE checklist is best used in conjunction with this article (freely available on the Web sites of PLoS Medicine at http://www.plosmedicine.org/, Annals of Internal Medicine at http://www.annals.org/, and Epidemiology at http://www.epidem.com/). Information on the STROBE Initiative is available at www.strobe-statement.org.

The report according to the GRABDROP guidelines of JoGH

1. Please list all papers published by each co-author in previous three years that were based on secondary analysis of a big data repository

Regarding research using data from the 2020 National Occupational Disease Surveillance Platform, published articles include:

(1) The relative contribution of PM2.5 components to the obstructive ventilatory dysfunction—insights from a large ventilatory function examination of 305,022 workers in southern China. https://doi.org/10.1016/j.envint.2024.108721

(2) Are workers also vulnerable to the impact of ambient air pollution? Insight from a large-scale ventilatory exam.
https://doi.org/10.1016/j.scitotenv.2024.174634

(3) The Impact of Blood Lead and Its Interaction with Occupational Factors and Air Pollution on Hypertension Prevalence.
doi: 10.3390/toxics12120861

2. Please explain the key elements of your study design and the use of the available datasets that make your study an original scientific contribution

First, our study focuses specifically on a working population, which represents a cohort that has not been a primary focus in prior research examining the association between air pollutants and anaemia. Second, the data from this surveillance platform were accessed exclusively by members of our research team for this project, and there is no overlap or duplication in the research topics being investigated. Therefore, the risk of duplicate publication resulting from multiple analyses of the same dataset is effectively mitigated. Third, our study innovatively examines the potential modifying role of occupational variables in the relationship between air pollution and anaemia. Collectively, these elements substantiate that our work constitutes an original scientific contribution.

3. Please list all publications that addressed similar research questions in the same dataset and indicate where you cited them in your paper

Due to the restricted nature of data access from this platform, other research teams are precluded from conducting similar analyses on the identical dataset. Accordingly, no similar publications using this specific dataset have been cited in this manuscript.

4. **Please explain how you addressed multiple testing through an appropriately rigorous statistical threshold and indicate this in the methods section**

To address issues related to multiple testing, we explicitly stated the exploratory nature of the subgroup analyses and emphasized cautious interpretation of the findings rather than relying solely on statistical significance. We would like to clarify that all subgroup analyses in our study were conceived and interpreted as exploratory and hypothesis-generating rather than confirmatory. Their primary aim was hypothesis-generating, to identify potential patterns that might warrant further investigation in future studies, rather than to draw definitive conclusions. Consistent with the approach taken in several prior high-impact studies in this field (Elser H et al.,2025; Gao Q et al., 2025; Aron, J et al., 2024; Di et al., 2017), we presented these analyses without multiplicity adjustment to explore which subgroups might exhibit a stronger association between air pollution and anemia. We have provided corresponding supplementary notes in the Statistical Analysis section and the section "Potential modifiers of the association between air pollution and Hb and anaemia" of

the Discussion.

(1) Elser H, Frankland TB, Chen C, Tartof SY, Mayeda ER, Lee GS, et al. Wildfire Smoke Exposure and Incident Dementia. JAMA Neurol. 2025;82:40–8. Medline:39585704 doi:10.1001/jamaneurol.2024.4058

(2) Gao Q, Jiang B, Tong M, et al. Effects and interaction of humidex and air pollution on influenza: A national analysis of 319 cities in mainland China. J Hazard Mater. 2025;490:137865. doi:10.1016/j.jhazmat.2025.137865

(3) Aron, J., Baldomero, A. K., Rau, A., Fiecas, M. B., Wendt, C. H., & Berman, J. D. (2024). Individual Risk Factors of PM2.5 Associated With Wintertime Mortality in Urban Patients With COPD. Chest, 165(4), 825–835. https://doi.org/10.1016/j.chest.2023.10.016

(4) Di, Q., Wang, Y., Zanobetti, A., Wang, Y., Koutrakis, P., Choirat, C., Dominici, F., & Schwartz, J. D. (2017). Air Pollution and Mortality in the Medicare Population. The New England Journal of Medicine, 376(26), 2513–2522. https://doi.org/10.1056/NEJMoa1702747

5. Please declare to what extent have AI chatbots been used in developing your paper and to which parts of the paper did they contribute

AI was only used strictly for linguistic polishing to improve the readability and coherence of the text.
